# Parallel Mapping and Simultaneous Sequencing Reveals Deletions in *BCAN* and *FAM83H* Associated with Discrete Inherited Disorders in a Domestic Dog Breed

**DOI:** 10.1371/journal.pgen.1002462

**Published:** 2012-01-12

**Authors:** Oliver P. Forman, Jacques Penderis, Claudia Hartley, Louisa J. Hayward, Sally L. Ricketts, Cathryn S. Mellersh

**Affiliations:** 1 Kennel Club Genetics Centre, Animal Health Trust, Kentford, United Kingdom; 2 The School of Veterinary Medicine, University of Glasgow, Glasgow, United Kingdom; University of Bern, Switzerland

## Abstract

The domestic dog (*Canis familiaris*) segregates more naturally-occurring diseases and phenotypic variation than any other species and has become established as an unparalled model with which to study the genetics of inherited traits. We used a genome-wide association study (GWAS) and targeted resequencing of DNA from just five dogs to simultaneously map and identify mutations for two distinct inherited disorders that both affect a single breed, the Cavalier King Charles Spaniel. We investigated episodic falling (EF), a paroxysmal exertion-induced dyskinesia, alongside the phenotypically distinct condition congenital keratoconjunctivitis sicca and ichthyosiform dermatosis (CKCSID), commonly known as dry eye curly coat syndrome. EF is characterised by episodes of exercise-induced muscular hypertonicity and abnormal posturing, usually occurring after exercise or periods of excitement. CKCSID is a congenital disorder that manifests as a rough coat present at birth, with keratoconjunctivitis sicca apparent on eyelid opening at 10–14 days, followed by hyperkeratinisation of footpads and distortion of nails that develops over the next few months. We undertook a GWAS with 31 EF cases, 23 CKCSID cases, and a common set of 38 controls and identified statistically associated signals for EF and CKCSID on chromosome 7 (P_raw_ 1.9×10^−14^; P_genome_ = 1.0×10^−5^) and chromosome 13 (P_raw_ 1.2×10^−17^; P_genome_ = 1.0×10^−5^), respectively. We resequenced both the EF and CKCSID disease-associated regions in just five dogs and identified a 15,724 bp deletion spanning three exons of *BCAN* associated with EF and a single base-pair exonic deletion in *FAM83H* associated with CKCSID. Neither *BCAN* or *FAM83H* have been associated with equivalent disease phenotypes in any other species, thus demonstrating the ability to use the domestic dog to study the genetic basis of more than one disease simultaneously in a single breed and to identify multiple novel candidate genes in parallel.

**Authors Summary**

The Cavalier King Charles Spaniel (CKCS) is popular as a companion breed of dog in many countries worldwide. However, in common with other breeds, it is documented to suffer from a high frequency of inherited disorders, which are largely the result of routine breeding practices. The homogeneous population structure of individual breeds is advantageous for mapping inherited conditions, and we sought to utilise this by mapping two disorders, episodic falling (EF) and congenital keratoconjunctivitis sicca and ichthyosiform dermatosis (CKCSID), using a genome-wide association study approach comprising a set of cases for each condition and a single set of common controls. Independent disease-associated regions were identified for EF and CKCSID, both containing approximately 100 genes. In the absence of any provocative candidate genes, we resequenced both entire regions simultaneously using two cases for each disease and one clinically unaffected control. A 15.7 kb deletion in the *BCAN* gene was associated with EF and a 1 bp deletion in *FAM83H* was associated with CKCSID. Neither gene has been associated with similar conditions previously. This investigation highlights how multiple disease-associated mutations can be simultaneously identified in the dog with a minimal set of individuals.

## Introduction

The domestic dog is well documented as being an excellent model for studying the genetics of both simple and complex traits [1]–[3]. The purebred dog population comprises several hundred distinct breeds that usually originate from small numbers of founding individuals, with each breed representing an isolated and relatively homogenous closed breeding population. Within breeds there is strong selection for desired phenotypic and behavioural characteristics, and dogs that possess these desired characteristics are often bred with extensively. The resulting population structure of individual breeds can result in the propagation of many spontaneously occurring deleterious mutations within a breed and these can exist at high frequencies, with the possibility of multiple inherited disorders arising within a single breed. A large proportion of these disorders show a Mendelian autosomal recessive mode of inheritance. The Staffordshire Bull Terrier and Labrador Retriever are two of the many examples of breeds that are affected by at least two autosomal recessive disorders for which the causal mutations have been identified; hereditary cataract and the metabolic disorder L-2-hydroxyglutaric aciduria in the Staffordshire Bull Terrier [4]–[5] and exercise induced collapse and centronuclear myopathy in the Labrador Retriever [6]–[7]. The high levels of homogeneity and long linkage disequilibrium that are characteristic of the genomes of most purebred dog breeds means that canine disease traits can be mapped using much smaller numbers of cases and controls and far more modest numbers of genetic markers than would be required to map an equivalent disease in outbred human populations [8]–[9].

Episodic falling (EF) in the Cavalier King Charles Spaniel (CKCS) was first reported in 1983, although the condition has been recognised in the breed since at least the early 1960s [10]. EF (also known within the breed as “sudden collapse”, muscle hypertonicity and hyperekplexia) is a paroxysmal exertion-induced dyskinesia that is usually exercise, excitement or stress-induced and is characterised by muscular hypertonicity and abnormal posturing, with affected dogs appearing to demonstrate a temporary inability to relax the affected limb and trunk muscles [10]. EF in the CKCS has an age of onset of between three to seven months of age, and affects both male and female dogs. Pedigree inspection indicates that the disease has an autosomal recessive mode of inheritance. The episodes are usually brief (less than five minutes) and self-limiting, but the clinical signs between cases are variable, with the length of an episode ranging from a few seconds to several minutes. Episodes start with an increase in muscle tone, with bunny-hopping movements [10] and/or presence of a “deer stalker” gait [11]. The back may become arched and the head held close to the ground leading to collapse, either to the side or forwards. Legs may be held out in a rigid, extended fashion, although in some cases the dog may return to the feet within seconds of a collapse. In some of the most severe cases, forelegs or hind legs become protracted until they are positioned over the top of the dog's head. Dogs appear to remain fully conscious during an episode [10].

EF in the CKCS shares similarities to human disorders, including idiopathic (familial and sporadic) paroxysmal exertion-induced dyskinesia, hyperekplexia, Brody's myopathy, and myotonia. Paroxysmal dyskinesias comprise a spectrum of conditions in human patients, all of which are characterised by involuntary movements triggered by specific events. Paroxysmal exertion-induced dyskinesia is a paroxysmal dyskinesia that is induced by prolonged exercise of between 15 to 60 minutes duration. Patients are variably affected, with episodes lasting from minutes to a couple of hours. Episodes are usually restricted to those muscle groups that have been exercised. The response to treatment is poor, although some limited benefit has been described using anticonvulsant medications [12]. Hyperekplexia is a disease of exaggerated startle response and increased muscle stiffness and rigidity which shows a particularly close resemblance to EF in terms of the positive response to the drug clonazepam. Clonazepam is thought to improve neurotransmission in gamma-aminobutyric acid (GABA) inhibitory pathways [13]. The genes *GLRA1*, *GLRB*, *SLC6A5*, and *GPHN*, which all encode proteins involved in glycinergic neurotransmission, have been associated with hyperekplexia in humans [14]–[17]. Brody's myopathy is a disease of exercised-induced muscle cramping with the inability to relax muscles [18]. Mutations in the gene *SERCA1* and *ATP2A1* have been associated with Brody's myopathy [19]. Myotonia is described as a disease with delayed skeletal muscle relaxation after sudden and often exaggerated contraction. Myotonia exists in both autosomal recessive (Becker's disease) and dominant forms (Thomsen's disease), and is caused by mutations in the *CLCN1* gene [20]–[21]. Mutations in *CLCN1* have been associated with myotonia in Miniature Schnauzer and the Australian Cattle Dog [22]–[23].

A distinct, congenital condition in the CKCS that affects the skin, eyes and nails is the syndrome known as congenital keratoconjunctivitis sicca and ichthyosiform dermatosis (CKCSID), or more commonly referred to as “dry eye curly coat syndrome”. This condition, which was first reported in the scientific literature in 2006 [24], manifests at birth, with further clinical signs evident in early life. Cases present with a congenitally abnormal (rough/curly) coat, signs of keratoconjunctivitis sicca (KCS) from eyelid opening, and are usually smaller than littermates. Reduced production of aqueous tears and tear film qualitative abnormalities result in a tacky mucoid or mucopurulent ocular discharge and ulceration of the cornea in severe cases. Persistent scale along the dorsal spine and flanks with a harsh, frizzy and alopecic coat is evident in the first few months of life, often causing the dog to scratch. Ventral abdominal skin becomes hyperpigmented and hyperkeratinised in adulthood. Footpads are hyperkeratinised from young adulthood with nail growth abnormalities and intermittent sloughing, causing pain and lameness. Affected dogs also tend to suffer increased dental disease, some dogs requiring tooth extraction [24]. Examination of the oral cavities of affected adult dogs revealed extensive tartar formation with associated gingivitis, particularly of premolar and molar teeth, in most cases. Frequently, single or multiple tooth extraction had been undertaken if dogs had received dental treatment under general anaesthesia. Tooth enamel, however, was not grossly abnormal. Disease management is difficult, with many owners opting to euthanize affected puppies on welfare grounds. No other occurrences of combined KCS and ichthyosiform dermatosis have been reported in any other breeds in the veterinary literature, although mutations associated with ichthyosis have been identified in the Norfolk Terrier and Jack Russell Terrier [25]–[26]. Although no human conditions have been described that closely resemble CKCSID, keratitis-ichthyosis-deafness (KID) syndrome shares more than one similar clinical sign, and is caused by mutations in the *GJB2* gene encoding connexin-26 [27]–[28]. A syndrome of woolly hair, premature tooth loss, nail dystrophy, acral hyperkeratosis and facial abnormalities has also been described in a human kindred, but no ocular clinical signs were reported [29].

The aim of our investigation was to identify the mutations associated with EF and CKCSID in the CKCS using a genome-wide association study (GWAS) mapping approach, followed by targeted resequencing to identify the causal variant underlying the association signal for each disease. We sought to capitalise on the fact that both conditions affect the same breed by using a common set of controls for both the EF and CKCSID association studies and a minimal number of dogs for resequencing and mutation identification.

## Results

**Figure 1:**
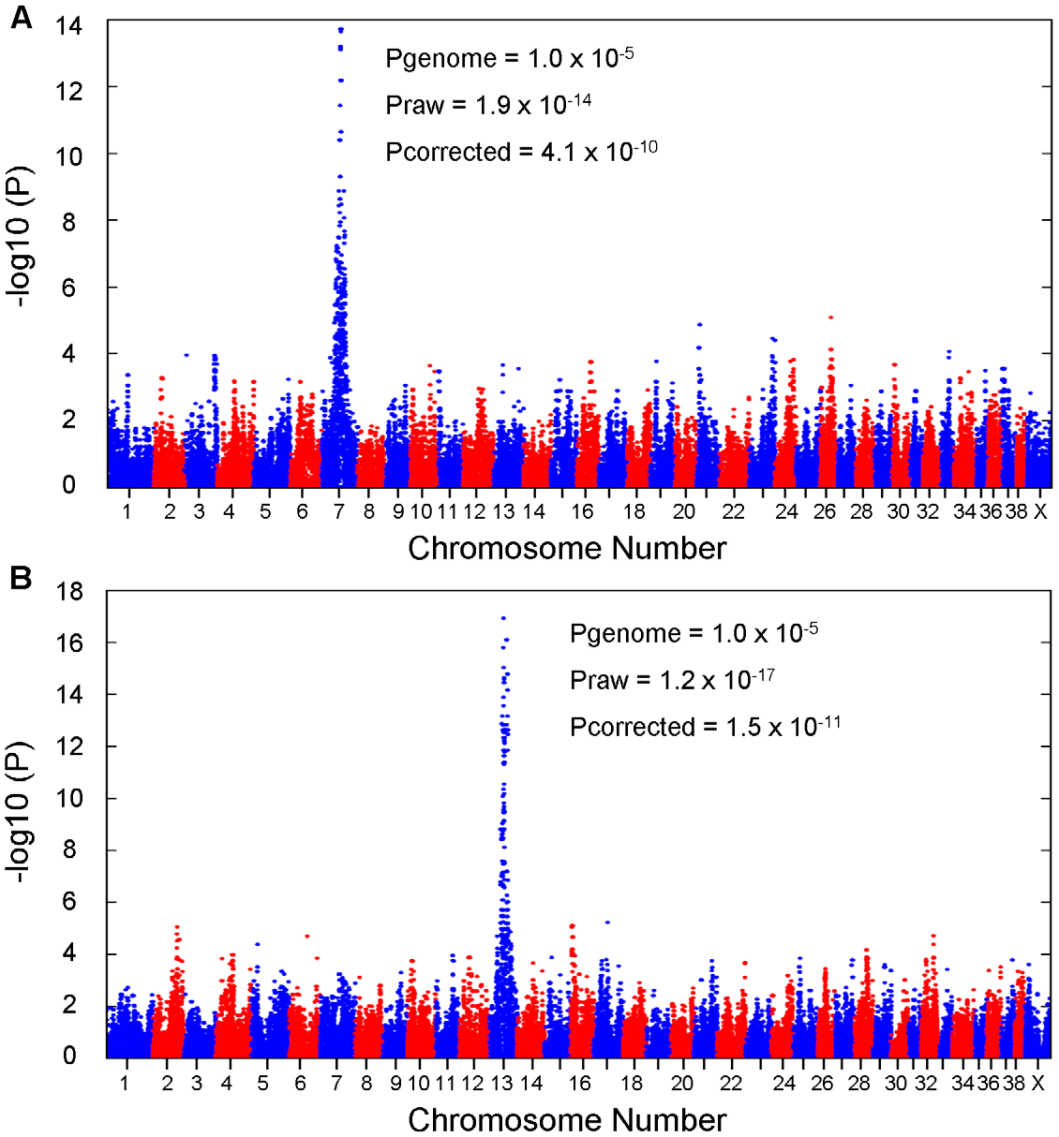
Results of genome-wide association studies. For (A) EF and (B) CKCSID.

We conducted GWAS analyses for each disease independently using 31 EF cases and 19 CKCSID cases aligned to a common set of 38 controls (unaffected for both conditions). Association analyses revealed a single strong statistical signal for EF on chromosome 7 (P_raw_ 1.9×10^−14^ ; P_genome_ = 1.0×10^−5^) and for CKCSID on chromosome 13 (P_raw_ 1.2×10^−17^ ; P_genome_ = 1.0×10^−5^ ) (Figure 1). Genomic inflation values based on the median chi-squared were 1.57 and 1.62 for the EF and CKCSID association analyses respectively. This level of inflation of test statistics is commonly observed in within-breed GWAS in the purebred dog due to population substructure and high levels of relatedness among individuals. We therefore conducted additional association analyses to attempt to adjust for these effects using a mixed model approach implemented in the statistical package R [30]. The two top association signals remained statistically associated at 4.1×10^−10^ and 1.5×10^−11^ for the EF and CKCSID respectively. QQ plots are displayed for the corrected data in Figure S1.

To identify regions of shared homozygosity between EF and CKCSID cases, we visually examined individual SNP genotypes spanning the two disease-associated regions. The positions of recombination events in cases, resulting in loss of shared homozygosity, were used to define the disease-associated critical haplotype for EF (chromosome 7: 44,093,554 bp to 47,048,914 bp) and for CKCSID (chromosome 13: 39,648,169 bp to 42,481,707 bp) based on the CanFam2.0 reference genome. Genes in the disease-associated regions and synteny to the human chromosomes are displayed in Figures S2 and S3. There were six EF cases that were not homozygous for the chromosome 7 disease-associated haplotype. Of these six cases, five were owner reported cases. The remaining discordant case had been diagnosed with EF by a veterinary neurologist, but during a subsequent consultation the dog was re-assessed and its diagnosis was changed to one of primary epilepsy. All CKCSID cases were homozygous for the same disease-associated haplotype.

**Figure 2:**
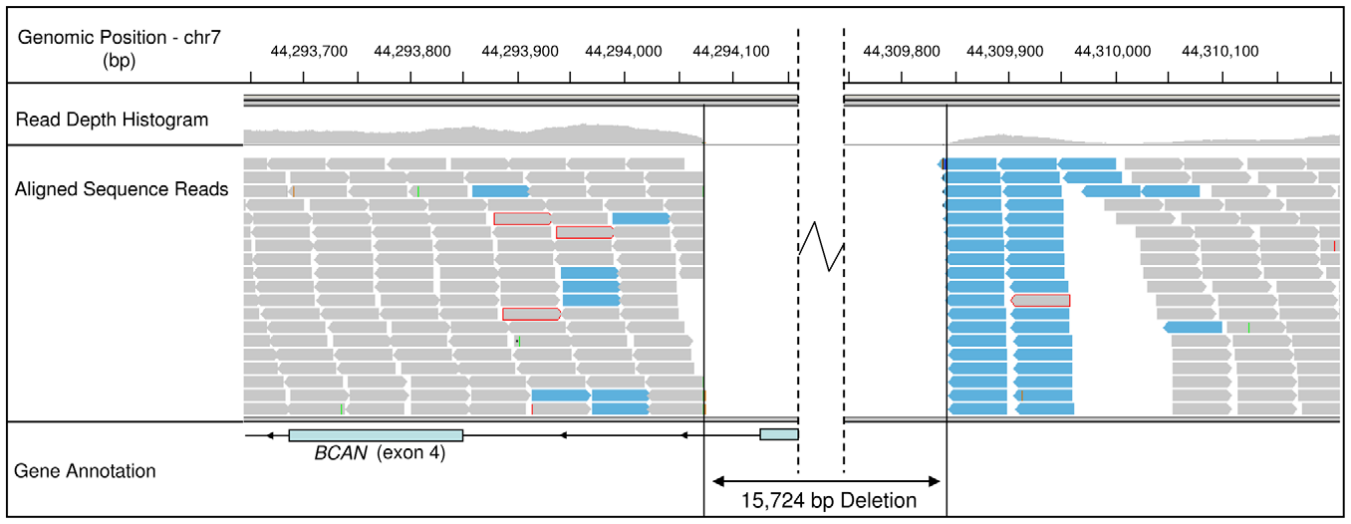
Sequence read alignments for an EF case, spanning the 5′ region of the *BCAN* gene. Grey bars represent correctly aligned sequence reads. Reads with a red perimeter indicate that the mate has not been aligned for the read pair. Blue bars represent reads that have been flagged for inconsistent insert size; indicative of a genomic deletion.

**Figure 3:**
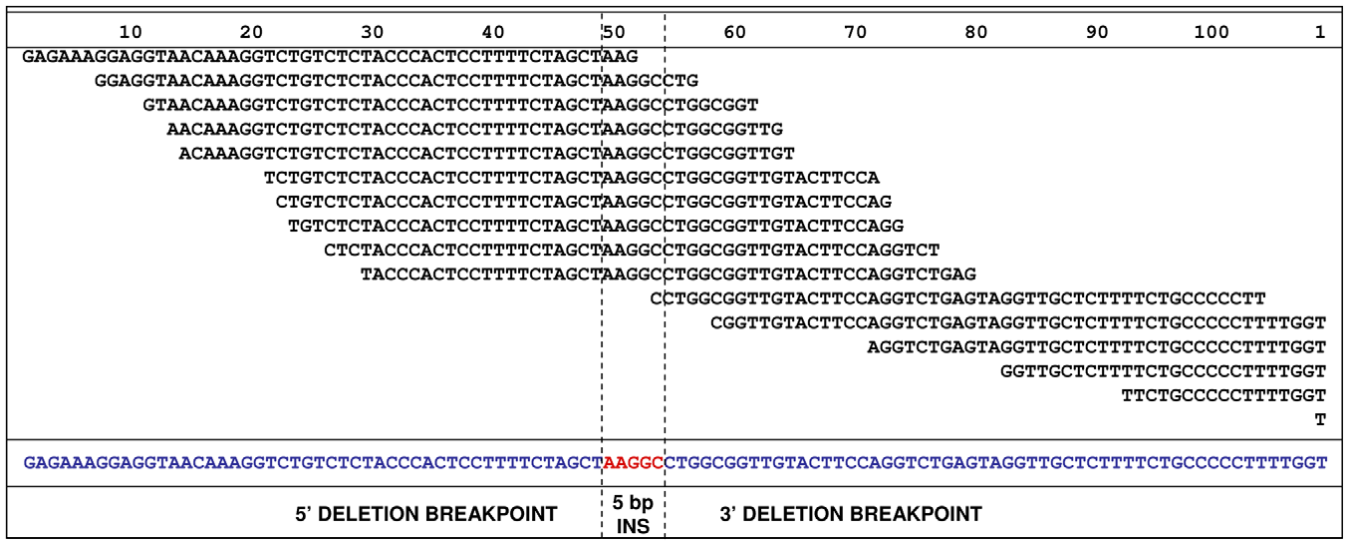
*De novo* assembly of reads, from a single EF case, aligned across the deletion.

**Figure 4:**
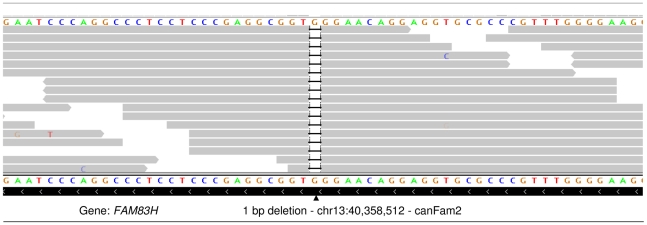
IGV display of the 1 bp deletion in exon 5 of *FAM83H*.

**Figure 5:**
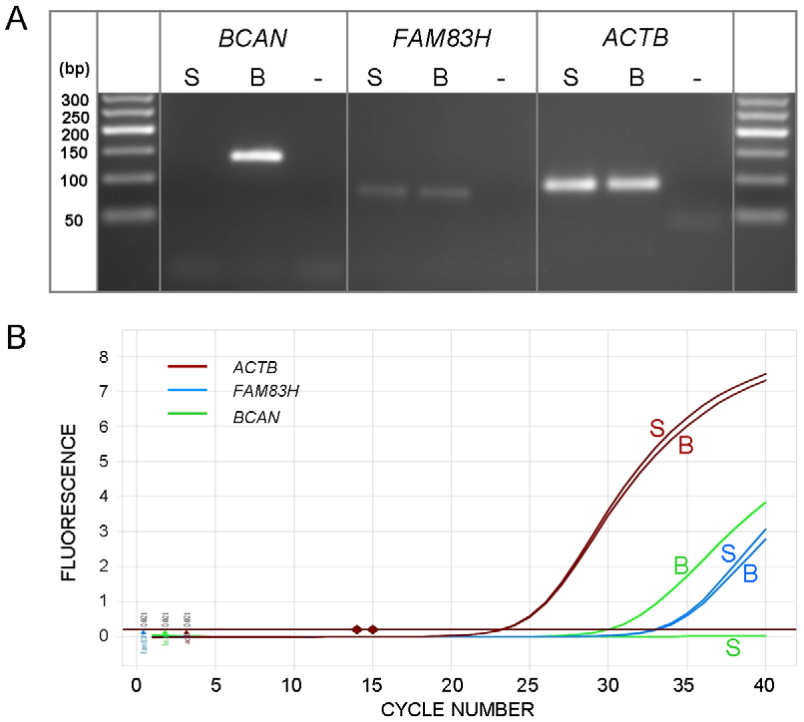
Expression levels of *BCAN* and *FAM83H*. Expression levels in brain (B) and skin (S) relative to the reference gene *ACTB*. PCRs were assessed at end point by agarose gel electrophoresis (A) and in real-time by qRT-PCR (B). Reactions performed in triplicate, but displayed as single curves for illustrative purposes.

To attempt to identify the causal variants underlying the association signals for each disease, we used a targeted resequencing approach to resequence both the EF and the CKCSID critical intervals of association in two EF cases, two CKCSID cases, and one unaffected control. Individuals for sequencing were based on specific haplotypes. Combined, the disease-associated critical intervals spanned genomic regions totalling 5,788,898 bp. Sequencing coverage was limited to approximately 65% of the target regions, as repetitive DNA elements accounting for ∼35% of the target regions were masked during target enrichment probe design. Sequence reads were aligned to the canine reference genome, and SNP and indel calls were made and collated into a single file. The aligned sequence reads were analysed for structural variants by visual inspection and read depth comparison. We identified ∼8700 SNPs and ∼1400 indels across the targeted region per dog sequenced, in comparison to the canine reference genome. We also identified deletions of ∼6 kb, ∼10 kb and ∼16 kb, only the latter of which spanned a coding region. In the EF disease-associated region, two provocative mutations were identified that segregated with EF in the five dogs sequenced. These were a single base mutation in the *DENN/MADD domain containing 4B* gene (*DENND4B*) causing an arginine to a histidine amino acid substitution, and a ∼16 kb deletion encompassing the first three exons of the *brevican* gene (*BCAN*) (Figure 2). Both polymorphisms were further investigated by genotyping the full panel of cases and controls used in the GWAS. The *DENND4B* mutation did not segregate as strongly with EF as the *BCAN* deletion and was thus excluded. In addition, the *DENND4B* mutation was identified in three non-CKCS dogs (two Italian Spinoni and one Golden Retriever) genotyped on the CanineHD chip as array controls, so is likely to be a common canine polymorphism. Full segregation analysis data is shown in Table S1. Exact deletion breakpoints of the *BCAN* deletion were defined by *de novo* assembly of the reads aligned across and surrounding the deletion breakpoints using Gap4 (Staden Package) [31]. *De novo* assembly revealed the deletion to be 15,724 bp with a small insertion of 5 bp spanning the deletion breakpoints that is not present in control dogs (Figure 3). In the CKCSID disease-associated genomic region only a single provocative mutation was identified that segregated appropriately with CKCSID in the five dogs that were sequenced. This was a single base deletion in exon 5 of the gene *family with sequence similarity 83*, *member H* (*FAM83H*) (Figure 4). Buccal epithelia and brain (cerebellum) cDNA sequencing confirmed the exon boundaries of the *FAM83H* and *BCAN* genes respectively (Genbank accession numbers JN968466–JN968467). The *BCAN* deletion spans exons 1–3 and is potentially a complete gene knockout. The *FAM83H* single base deletion is in exon 5, and is predicted to truncate the peptide from 1151 to 582 amino acids, with 257 aberrant amino acids at the C terminal. Quantitative reverse transcription PCR (qRT-PCR) was used to assess *FAM83H* and *BCAN* expression levels in canine skin and brain (cerebellum) tissues. *BCAN* expression was confirmed in the brain, but was not detected in the skin. A similar level of *FAM83H* expression was detected in both skin and brain (Figure 5). *FAM83H* expression was also detected in footpad and buccal epithelia by RT-PCR (data not shown).

To further validate the associations between the *BCAN* and *FAM83H* deletions and EF and CKCSID respectively, we genotyped a panel of 308 CKCS for both variants. This panel included the 31 EF cases, 19 CKCSID cases and 38 controls used in the GWAS analyses, and an additional 17 EF cases, 5 CKCSID cases and 198 controls. Results are shown in Table 1. In addition a panel of 341 dogs from 34 other breeds (with at least 2 dogs per breed) were assayed for both the *FAM83H* and *BCAN* mutations. All 341 dogs were homozygous wild-type for both polymorphisms. From the panel of 308 CKCS genotyped for the *BCAN* or *FAM83H* mutations, individuals that were not clinically affected, unrelated at the parent level and not related to cases at the parent level were used to estimate the mutation frequencies in the UK. From these 122 individuals, the allele frequency of both variants was estimated at 0.08.

**Table 1:** Summary of genotyping results on the panel of 308 dogs assayed for the *BCAN* ∼16 kb deletion and the *FAM83H* 1 bp deletions.

	*BCAN* Genotype		
	(−/−)	(−/wt)	(wt/wt)
EF cases	39	3	6
EF controls	17	62	181

	*FAM83H* Genotype		
	(−/−)	(−/wt)	(wt/wt)
CKCSID cases	24	0	0
CKCSID controls	0	38	246

Homozygous mutants are shown in column (−/−), heterozygous dogs in column (−/wt) and homozygous wild type dogs in column (wt/wt).

## Discussion

Using a genome-wide association study approach followed by targeted resequencing, we have identified mutations in *BCAN* and *FAM83H* associated with two genetically distinct autosomal recessive conditions in the CKCS. We capitalised on the fact that both diseases occurred in a single breed of dog, by using a single set of common controls aligned to both case sets for the EF and CKCSID association studies, and by using just five dogs to resequence both disease-associated critical regions simultaneously. This allowed the investigation to take place with a modest sample set, and proved to be a highly efficient and effective method to identify in parallel two separate disease-associated variants in the CKCS. The fact that we were able to use resequenced EF cases as additional controls for CKCSID and vice-versa enabled us to effectively filter sequence variants to limit the number of candidate causal variants for follow-up.

No mutant *BCAN* or *FAM83H* alleles were detected among 341 dogs from 34 other breeds. This suggests that the two mutations are limited to the CKCS breed, although only a small selection of dogs was tested from just a subset of all dog breed populations. Additional dogs would need to be screened to formally conclude that the mutation is not present in any other breeds.

The EF-associated gene *BCAN* encodes brevican, which is one of the central nervous system specific members of the hyaluronan-binding chondroitin sulfate proteoglycan family [32]. Brevican is important in the organisation of the nodes of Ranvier in myelinated large diameter axons [33] and disruption of this region results in a delay in axonal conduction [34]. Interestingly the gene *HAPLN2* is tandomly arranged upstream of *BCAN*. *HAPLN2* encodes Bral1, a brain specific hyaluronan and protoglycan link protein and is co-localised with brevican and versican V2 to form complexes at the nodes of Ranvier [34]. The BCAN deletion moves the 3′ UTR of *HAPLN2* to within 2 kb of exon 4 of *BCAN*. Expression analysis would be required to fully establish whether the ∼16 kb deletion causes a complete knock-out of the BCAN gene and to investigate any potential effects on HAPLN2 expression.

Mutations in *BCAN* have not previously been associated with a disease phenotype and brevican-deficient mice are viable, fertile, physiologically normal, display normal behaviour and have a normal life expectancy [35]. However, the absence of any apparent abnormalities in brevican-deficient mice may relate to an absence of episode triggers within the environment the mice were maintained in. In dogs, episodes are induced by exercise or excitement and it is highly likely that mice will not exercise sufficiently intensely within their routine laboratory environment. EF has a variable phenotype in the CKCS and 17 out of 56 dogs that were homozygous for the *BCAN* deletion were reportedly not affected by EF, which suggests that the disorder may be influenced by variation in environmental stimuli and potential variants in modifier genes. As EF is an exercise-induced condition, differences in levels of activity among affected dogs may partially account for some of the phenotypic variation. One dog in our study that was homozygous for the *BCAN* mutation but did not display clinical signs consistent with EF was reported by its owner to be “docile and unexcitable”, suggesting, for this dog at least, insufficient environmental stimuli were provided to trigger the condition. In addition, nine out of 48 EF cases were not homozygous for the *BCAN* mutation. An extensive neurological assessment would be required to mitigate against misclassification of these cases, but this is often not possible for canine patients due to expense or lack of owner consent. The CKCS breed is also affected by idiopathic epilepsy, and the EF episodes may often be difficult to distinguish from epileptic seizures, as a definitive diagnostic test is not available for either condition [36]. It is therefore possible that for some cases epilepsy or other neurological conditions have wrongly been diagnosed as EF, although it is formally possible that there may be a second, genetically distinct form of EF in the CKCS. We did not completely resequence the entire association interval due to the repetitive nature of around 35% of the sequence, and although unsequenced regions were largely non-coding, potential causal mutations could be situated within these regions and would not therefore have been identified in our current study.

EF is a condition that becomes self-limiting and can self-rectify in some cases, with some dogs becoming clinically normal after a period of months to years of being clinically affected. It is interesting to speculate that this might be due to compensatory effects of other chondroitin sulphate proteoglycans in the brain, in particular versican V2 [33] taking over the role of brevican, although the effect could also be due to modified owner and/or dog behaviour in response to the episodes, such as a change in exercise levels or the avoidance of trigger events once these have been identified.

An identical mutation in *BCAN* has recently been associated with EF in the CKCS, by an independent research group, and the finding published whilst this manuscript was under review [37].

Several mutations in the CKCSID-associated gene *FAM83H* have been associated with autosomal-dominant hypocalcification amelogenesis imperfecta (ADHAI) in humans, which is a disease of faulty tooth enamel formation [38]. To date the mutations associated with ADHAI have all been found in exon 5 of *FAM83H* and are either nonsense or frameshift mutations leading to a premature stop codon after a sequence of aberrant amino acids. Further to this, mutations in the 5′ region of exon 5 appear to result in a generalised phenotype, affecting all teeth, compared to mutations occurring in the 3′ region, which appear to give a localised phenotype, with just a subset of teeth being affected [39]. The canine mutation is in a position which would predict a more generalised phenotype. Anecdotal evidence suggests that CKCSID cases do show clinical signs of tooth disease, although this is a post-hoc observation and would require further investigation to determine the exact nature of the dental problems.

The CKCSID phenotype suggests that *FAM83H* has an important role in skin development and regulation, in addition to enamel formation, at least in the dog. Limited expression analysis has revealed that *FAM83H* is expressed in canine skin, and also in the brain (cerebellum), footpad and buccal epithelia, in concordance with previous reports that *FAM83H* may be ubiquitously expressed [38]. Species-specific differences in gene expression and function have not currently been investigated and no significant skin or nail phenotypes have been associated with ADHAI in human patients. In humans all *FAM83H* mutations reported to date have been dominant, and no human patients with homozygous *FAM83H* mutations have been reported. In contrast the canine mutation is recessive and heterozygous dogs do not have a discernable phenotype, so it is interesting to speculate that the gene is playing a different role in enamel formation between the two species and that human patients may present additional phenotypes, similar to CKCSID, if a deleterious homozygous *FAM83H* mutation was identified.

In summary, we have identified mutations in two genes that are associated with distinct autosomal recessive disorders in the CKCS, neither of which have been previously associated with similar disease phenotypes in other species. The discovery of these mutations may suggest potential novel biological functions for *FAM83H* and *BCAN*, although formal proof of this would require further functional data to confirm the causality of the two mutations with respect to their associated disease phenotypes. This study illustrates how two disease phenotypes in a single dog breed can be investigated using a very modest sample set to successfully identify disease-associated mutations, using a GWAS approach followed by targeted resequencing.

## Materials and Methods

### Sample collection

All DNA samples were collected from CKCS in the general pet dog population. We collected residual blood samples that were drawn as part of a veterinary procedure or buccal swab samples that were collected by owners or by veterinarians. EF cases were defined as dogs reported by their owner and/or veterinarian to be displaying clinical signs consistent with EF, based on descriptions available in the scientific literature and in consultation with a veterinary neurologist. Video evidence was used where possible. In addition full neurological assessments were performed for some cases to rule out any other underlying causes. There is no definitive method of diagnosing EF, so diagnosis is based on the identification of a consistent clinical phenotype with the exclusion of other potential causes. All CKCSID cases included in the study were assessed by a single veterinary ophthalmologist. The additional panel of 341 dogs from 34 other breeds genotyped for both the *FAM83H* and *BCAN* mutations were all reported by their owners to be healthy and had been recruited to participate in other unrelated studies.

### DNA preparation

Genomic DNA was extracted from whole blood samples preserved in EDTA using the Nucleon BACC2 kit (Tepnel Life Science), or from buccal swabs using the QiaAmp Midi kit (Qiagen). Samples were concentrated using 0.5 ml Amicon Ultra 100 K centrifugal filter columns (Millipore) and normalised to 50 ng/µl after quantification on a Qubit fluorometer (Invitrogen).

### SNP genotyping and GWAS analyses

DNA samples from 31 EF cases, 19 CKCSID cases and 38 controls were genotyped using the Illumina CanineHD SNP genotyping array that comprises 173,662 SNPs (single nucleotide polymorphisms). The SNP genotyping dataset was analysed for association using the statistical package PLINK [40]. Sample call rates for all individuals were >99%. SNPs with a genotyping call rate of <95% and/or minor allele frequency of <5% were discarded. The strongest statistical signal from the unadjusted association analysis is termed P_raw_ (Figure 1). For the EF GWAS 91,427 SNPs were available for analysis following these quality control filters and for CKCSID 88,384 SNPs remained for analysis. Correction for multiple testing was performed using 100,000 MaxT permutations in PLINK. The strongest statistical signal after permutation is termed P_genome_ (Figure 1). Correction for population substructure and relatedness was performed using a mixed model, implemented in the statistical package R, and the strongest statistical signal is termed P_corrected_ (Figure 1) [30].

### Sequencing

Libraries were created for sequencing using next generation technology including the SureSelect solution based target enrichment stage (Agilent Technologies). RNA bait probes (120 bp) were designed to give 2× probe coverage of target regions using the online tool e-array (https://earray.chem.agilent.com/earray/). The total number of baits designed was 57,667 across 3.75 Mb of the 5.79 Mb target region (64.8% region coverage). The remaining 35.2% consisted of repeat regions that were masked during the bait design, or uncharacterised regions of the reference genome, and were thus not captured or resequenced.

Genomic DNA (5 µg) from two EF cases, two CKCSID cases and one control dog was used to prepare libraries for sequencing. DNA was fragmented by digesting with dsDNA Fragmentase (New England Biolabs) for 23 minutes at 37°C. Library fragments were end repaired, A-tailed and ligated to DNA adaptors for paired-end multiplexed sequencing (Illumina) using the NEBNext DNA Sample Prep Master Mix Set 1 (New England Biolabs). Oligonucleotides for adaptors and library amplification were manufactured by Integrated DNA Technologies. Pre-capture library amplification, sequence capture, and post-capture amplification was conducted using the SureSelect target enrichment system for Illumina paired-end multiplexed sequencing (Agilent Technologies). Libraries were quantified using the KAPA Library Quantification Kit (Kapa Biosystems). Paired-end sequencing (51 bp reads) was carried out on a single lane of an Illumina GAIIx at the Wellcome Trust Centre for Human Genetics, University of Oxford, UK. A 3.47 Gb dataset was produced giving an average read depth across target regions ranging from 95 to 114 for the five DNA libraries.

### Sequencing data analysis

Reads were aligned to the canine reference genome (CanFam2.0) using BWA [41]. SNP and indel calls were made using GATK [42]. Structural variant analysis was performed using Pindel [43]. Aligned reads were viewed using The Integrative Genomics Viewer (IGV) [44]. Polymorphisms occurring in exonic regions causing non-synonymous changes and in splice donor or acceptor sites were considered as candidate mutations. Candidate mutations were considered potentially as causal if they were homozygous mutant in cases and either heterozygous or homozygous wild-type in controls.

### RNA sequencing and qRT–PCR

RNA was extracted from post-mortem brain (cerebellum), skin and footpad tissues preserved in RNAlater (Life Technologies) and buccal epithelial tissue using a Qiagen RNeasy mini kit (Qiagen). cDNA was prepared using a Qiagen Quantitect reverse transcription kit. PCR was performed to confirm *BCAN* and *FAM83H* transcripts using 12 µl reactions consisting of 0.2 mM dNTPs (NEB), 1× PCR buffer (Qiagen), 0.5 µM forward primer, 0.5 µM reverse primer, 0.5 units HotStarTaq plus DNA polymerase (Qiagen) and ultrapure water. For PCR of GC-rich (>70%) regions, Q solution (Qiagen) was also added to the reaction at a final 1× concentration. Cycling parameters for PCR were 95°C for 10 minutes, followed by 35 cycles of 95°C for 30 seconds, 58°C for 30 seconds and 72°C for 60 seconds, and completed with a final elongation stage of 72°C for 10 minutes. PCR products were Sanger sequenced using Big Dye v3.1 (Applied Biosystems) for capillary electrophoresis on an ABI3130xl genetic analyser. Sequencing data were analysed using Gap4 (Staden package) [31]. Primers for cDNA sequencing are listed in Table S2. All primers were designed using Primer3 [45] and manufactured by IDT.

qPCR assays were carried out on an Illumina Eco machine in 10 µl reactions containing 5 µl Kapa Probe Fast qPCR mastermix, 1×IDT PrimeTime qPCR assay mix and 2 µl cDNA (primer sequences listed in Table S3). Reaction efficiencies were calculated using a seven point 2× serial dilution to create a standard curve. *BCAN*, *FAM83H* and *ACTB* reaction efficiencies were estimated at 97.5%, 95.7% and 94.3% respectively, with standard curve r^2^ values all >0.99.

### Genotyping of *FAM83H* and *BCAN* deletions

The 15,724 bp *BCAN* deletion and 1 bp deletion in *FAM83H* were assayed using PCR with fluorescently tagged primers. Primer sequences are listed in Table S4. The assay for the *BCAN* deletion used a three primer system and comprised a single fluorescently labelled forward primer 5′ of the deletion, paired with a reverse primer in the deleted region and a reverse primer 3′ of the deletion (Table S3). The *FAM83H* 1 bp deletion was assayed using a single primer pair, with the forward primer fluorescently labelled (Table S3). Assays were performed in multiplex PCR consisting of 1× PCR buffer, 1× Q solution, 200 µM dNTPs, 0.12 U Hotstar Taq plus, 0.3 µM CKCSID_F and CKCSID_R, 0.14 µM EF_F, 0.09 µM EF_bridge_R, 0.24 µM EF_normal_R and ultrapure water to a final volume of 12 µl. PCR cycling parameters were 95°C for 5 minutes, followed by 32 cycles of 95°C for 30 seconds, 58°C for 30 seconds and 72°C for 30 seconds, with a final elongation stage of 72°C for 10 minutes. PCR products were separated by capillary electrophoresis on ABI3130xl genetic analysers. Genotyping data were analysed using GeneMapper v4.0 (Applied Biosystems)

### Ethics statement

Collection of blood samples solely for research purposes is strictly monitored in the UK and requires a home office license. However any residual blood remaining after being drawn as part of a veterinary procedure may be used for research and does not require a license. Buccal swabbing is a relatively non-invasive procedure and does not require a license.

## Supporting Information

Figure S1 QQ plots of Fast Mixed Model corrected allelic association data for (A) EF and (B) CKCSID.
(TIF)

Figure S2 Graphical representation of genes in the EF disease associated genomic region and the syntenic region of the human genome, adapted from the Ensembl genome browser.
(TIF)

Figure S3 Graphical representation of genes in the CKCSID disease associated genomic region and syntenic regions of the human genome, adapted from the Ensembl genome browser.
(TIF)

Table S1 Genotype table for the *FAM83H* deletion (mutant allele designated 76), *BCAN* deletion (mutant allele designated 112) and *DENND4B* SNP. DNAs listed are the 96 samples that were genotyped in a single batch on the CanineHD SNP array, including the 31 EF cases, 19 CKCSIS cases, and 38 controls. Samples IDs marked * were excluded from the allelic association analysis. Sample IDs 2275, 5404 (Italian Spinoni) and 4748 (Golden Retriever) were genotyped for control purposes. Samples IDs 6804, 6836, 6888 and 15375 were CKCS ichthyosis cases. Sample ID 16837 was a genotyping outlier and therefore removed.
(DOC)

Table S2 Primers used for cDNA sequencing of the *FAM83H* and *BCAN* genes.
(DOC)

Table S3 Primers used for qPCR assays of the *BCAN*, *FAM83H* and *ACTB* (control) genes. All probes were 5′ 6-FAM and 3′ Iowa Black labelled, with internal ZEN labelling.
(DOC)

Table S4 Primers used in the genotyping assay for the *BCAN* and *FAM83H* mutations. The expected mutant product size for the CKCSID primer pair refers to the 1 bp deletion in the *FAM83H* gene and for the EF primer pairs refers to the 15,724 bp deletion encompassing the first three exons of *BCAN*.
(DOC)
