# Supplementary figures and images for: Parallel Mapping and Simultaneous Sequencing Reveals Deletions in BCAN and FAM83H Associated with Discrete Inherited Disorders in a Domestic Dog Breed
Source: PLoS Genet. 2012 Jan 12;8(1):e1002462. doi: 10.1371/journal.pgen.1002462 (PMC3257292; doi:10.1371/journal.pgen.1002462)

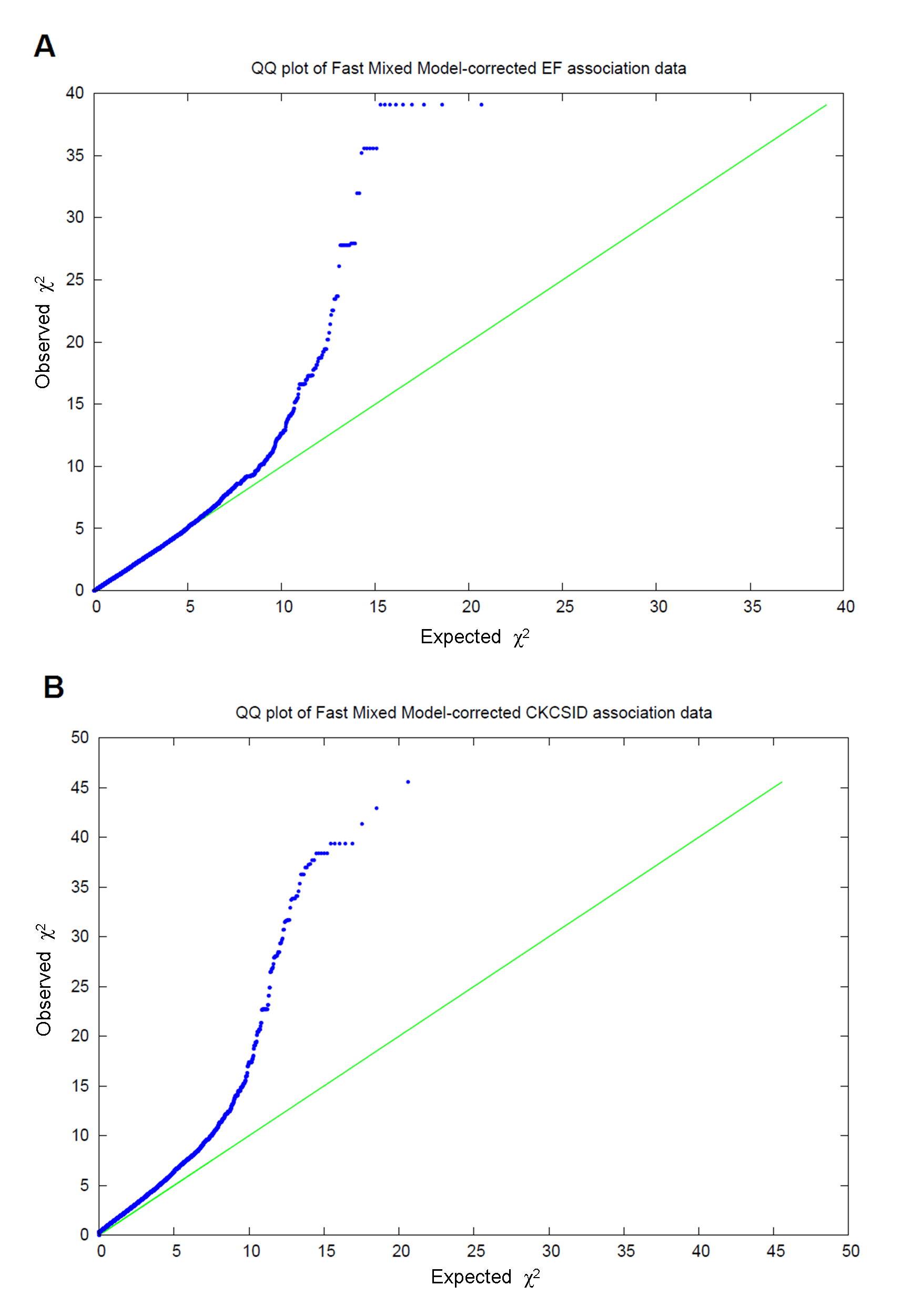

Supplement: Figure S1 — QQ plots of Fast Mixed Model corrected allelic association data for (A) EF and (B) CKCSID. (TIF) [file pgen.1002462.s001.tif]

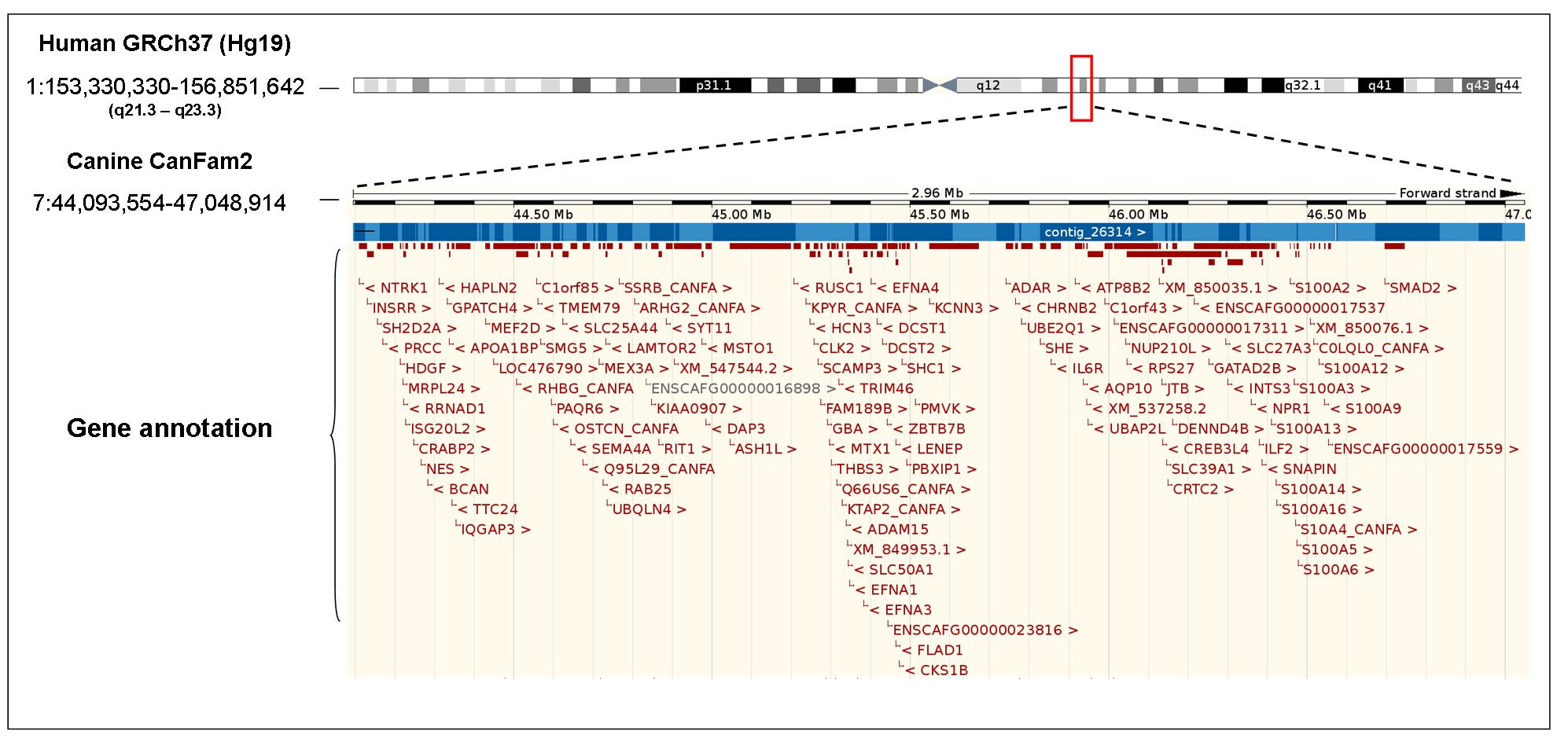

Supplement: Figure S2 — Graphical representation of genes in the EF disease associated genomic region and the syntenic region of the human genome, adapted from the Ensembl genome browser. (TIF) [file pgen.1002462.s002.tif]

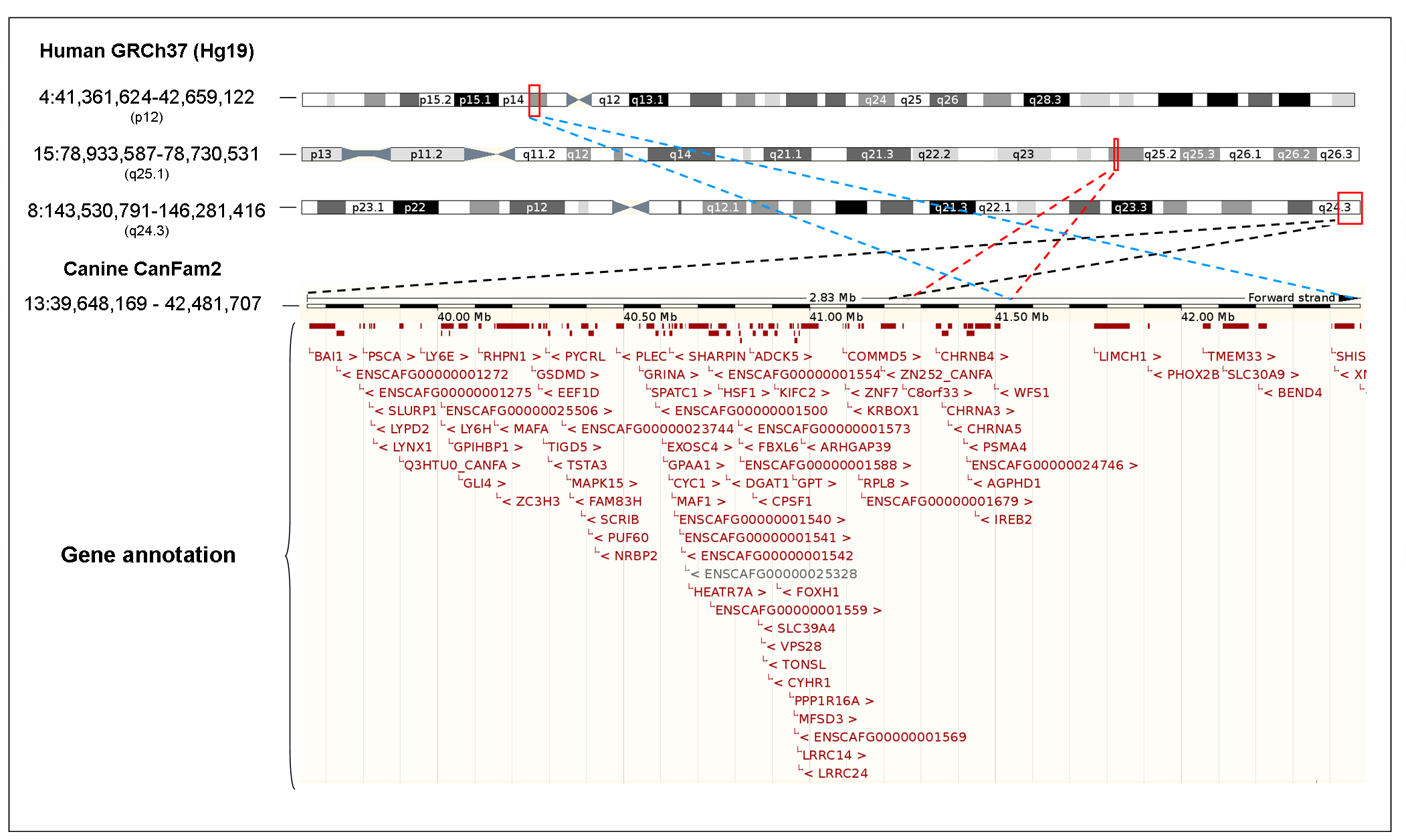

Supplement: Figure S3 — Graphical representation of genes in the CKCSID disease associated genomic region and syntenic regions of the human genome, adapted from the Ensembl genome browser. (TIF) [file pgen.1002462.s003.tif]
