# Supplementary material for: Parallel Mapping and Simultaneous Sequencing Reveals Deletions in BCAN and FAM83H Associated with Discrete Inherited Disorders in a Domestic Dog Breed
Source: PLoS Genet. 2012 Jan 12;8(1):e1002462. doi: 10.1371/journal.pgen.1002462 (PMC3257292; doi:10.1371/journal.pgen.1002462)
Supplement: Table S1 — Genotype table for the FAM83H deletion (mutant allele designated 76), BCAN deletion (mutant allele designated 112) and DENND4B SNP. DNAs listed are the 96 samples that were genotyped in a single batch on the CanineHD SNP array, including the 31 EF cases, 19 CKCSIS cases, and 38 controls. Samples IDs marked * were excluded from the allelic association analysis. Sample IDs 2275, 5404 (Italian Spinoni) and 4748 (Golden Retriever) were genotyped for control purposes. Samples IDs 6804, 6836, 6888 and 15375 were CKCS ichthyosis cases. Sample ID 16837 was a genotyping outlier and therefore removed. (DOC) [file pgen.1002462.s004.doc]

**Table S1**

|  |  |  | **Genotype** | | |
| --- | --- | --- | --- | --- | --- |
| **Sample ID** | **Breed** | **Status** | ***FAM83H*** | ***BCAN*** | ***DENND4B*** |
| 2275* | IS | CONTROL | 77, 77 | 106, 106 | A, A |
| 4748* | GR | CONTROL | 77, 77 | 106, 106 | G, A |
| 5404* | IS | CONTROL | 77, 77 | 106, 106 | A, A |
| 6804* | CKCS | CONTROL | 77, 77 | 106, 106 | G, G |
| 6823 | CKCS | CONTROL | 77, 77 | 106, 106 | G, G |
| 6835 | CKCS | CONTROL | 77, 77 | 106, 106 | G, G |
| 6836* | CKCS | CONTROL | 77, 77 | 112, 112 | A, A |
| 6888* | CKCS | CKCSID | 76, 76 | 106, 106 | G, G |
| 6975 | CKCS | CKCSID | 76, 76 | 106, 106 | G, G |
| 6979 | CKCS | CKCSID | 76, 76 | 106, 106 | G, G |
| 6981 | CKCS | CKCSID | 76, 76 | 106, 106 | G, G |
| 6987 | CKCS | CKCSID | 76, 76 | 106, 106 | G, G |
| 6988 | CKCS | CKCSID | 76, 76 | 106, 106 | G, G |
| 7086 | CKCS | CKCSID | 76, 76 | 106, 106 | G, G |
| 7190 | CKCS | CKCSID | 76, 76 | 106, 106 | G, G |
| 7859 | CKCS | CKCSID | 76, 76 | 106, 106 | G, G |
| 7875 | CKCS | CKCSID | 76, 76 | 106, 112 | G, A |
| 8600 | CKCS | CKCSID | 76, 76 | 106, 106 | G, G |
| 8601 | CKCS | CKCSID | 76, 76 | 106, 106 | G, G |
| 8710 | CKCS | CKCSID | 76, 76 | 106, 106 | G, G |
| 8711 | CKCS | CKCSID | 76, 76 | 106, 106 | G, G |
| 9915 | CKCS | CKCSID | 76, 76 | 112, 112 | A, A |
| 9916 | CKCS | CKCSID | 76, 76 | 106, 112 | G, A |
| 10709 | CKCS | CONTROL | 76, 77 | 106, 106 | G, G |
| 10835 | CKCS | CKCSID | 76, 76 | 106, 106 | G, G |
| 12433 | CKCS | EF | 77, 77 | 106, 112 | G, A |
| 12466 | CKCS | EF | 77, 77 | 112, 112 | A, A |
| 12552 | CKCS | CKCSID | 76, 76 | 106, 112 | G, A |
| 12823 | CKCS | CONTROL | 77, 77 | 106, 106 | G, G |
| 13055 | CKCS | CONTROL | 77, 77 | 106, 112 | G, A |
| 13056 | CKCS | CONTROL | 77, 77 | 106, 106 | G, G |
| 13071 | CKCS | CONTROL | 77, 77 | 106, 106 | G, G |
| 13088 | CKCS | CONTROL | 77, 77 | 106, 106 | G, G |
| 13102 | CKCS | CONTROL | 77, 77 | 106, 112 | G, A |
| 13137 | CKCS | EF | 77, 77 | 112, 112 | A, A |
| 13143 | CKCS | CKCSID | 76, 76 | 106, 106 | G, A |
| 13301 | CKCS | CONTROL | 76, 77 | 106, 106 | G, G |
| 13303 | CKCS | CONTROL | 76, 77 | 106, 106 | G, G |
| 13332 | CKCS | CONTROL | 77, 77 | 106, 106 | G, G |
| 13333 | CKCS | CONTROL | 77, 77 | 106, 106 | G, G |
| 13631 | CKCS | CONTROL | 77, 77 | 106, 106 | G, G |
| 13677 | CKCS | CONTROL | 77, 77 | 106, 106 | G, G |
| 13693 | CKCS | CONTROL | 77, 77 | 106, 106 | G, G |
| 13753 | CKCS | CONTROL | 77, 77 | 106, 106 | G, G |
| 13754 | CKCS | CONTROL | 76, 77 | 106, 106 | G, G |
| 13820 | CKCS | EF | 77, 77 | 112, 112 | A, A |
| 13821 | CKCS | CONTROL | 76, 77 | 106, 106 | G, G |
| 13850 | CKCS | CONTROL | 77, 77 | 106, 112 | G, A |
| 13917 | CKCS | CONTROL | 77, 77 | 106, 106 | G, G |
| 14174 | CKCS | CONTROL | 77, 77 | 106, 106 | G, G |
| 14216 | CKCS | CONTROL | 77, 77 | 106, 106 | G, G |
| 14225 | CKCS | CONTROL | 77, 77 | 112, 112 | A, A |
| 14228 | CKCS | CONTROL | 77, 77 | 106, 112 | G, A |
| 14306 | CKCS | CONTROL | 77, 77 | 106, 112 | G, A |
| 14339 | CKCS | CONTROL | 77, 77 | 106, 106 | G, G |
| 14511 | CKCS | CONTROL | 77, 77 | 106, 106 | G, G |
| 14512 | CKCS | CONTROL | 77, 77 | 106, 106 | G, G |
| 14740 | CKCS | CONTROL | 77, 77 | 106, 106 | G, G |
| 15375* | CKCS | CONTROL | 77, 77 | 106, 106 | G, A |
| 15552 | CKCS | CONTROL | 77, 77 | 106, 106 | G, G |
| 15943 | CKCS | EF | 77, 77 | 112, 112 | A, A |
| 16066 | CKCS | EF | 77, 77 | 106, 106 | G, G |
| 16067 | CKCS | EF | 77, 77 | 112, 112 | A, A |
| 16155 | CKCS | EF | 77, 77 | 112, 112 | A, A |
| 16642 | CKCS | EF | 76, 77 | 106, 106 | G, G |
| 16820 | CKCS | EF | 77, 77 | 112, 112 | A, A |
| 16821 | CKCS | EF | 77, 77 | 112, 112 | A, A |
| 16822 | CKCS | EF | 76, 77 | 112, 112 | A, A |
| 16823 | CKCS | EF | 77, 77 | 112, 112 | A, A |
| 16833 | CKCS | EF | 77, 77 | 112, 112 | A, A |
| 16837* | CKCS | CONTROL | 77, 77 | 106, 106 | A, A |
| 16843 | CKCS | CONTROL | 77, 77 | 112, 112 | A, A |
| 16852 | CKCS | EF | 77, 77 | 112, 112 | A, A |
| 16857 | CKCS | EF | - | - | - |
| 16861 | CKCS | EF | 77, 77 | 112, 112 | A, A |
| 16862 | CKCS | EF | 77, 77 | 112, 112 | A, A |
| 16864 | CKCS | EF | 77, 77 | 106, 106 | G, G |
| 16866 | CKCS | EF | 77, 77 | 112, 112 | A, A |
| 16867 | CKCS | EF | 77, 77 | 112, 112 | A, A |
| 16868 | CKCS | CONTROL | 77, 77 | 106, 112 | A, A |
| 16870 | CKCS | CONTROL | 77, 77 | 106, 106 | G, A |
| 16871 | CKCS | EF | 77, 77 | 112, 112 | A, A |
| 16874 | CKCS | CONTROL | 77, 77 | 112, 112 | A, A |
| 16876 | CKCS | EF | 77, 77 | 112, 112 | A, A |
| 16877 | CKCS | EF | 77, 77 | 112, 112 | A, A |
| 16878 | CKCS | CONTROL | 77, 77 | 106, 112 | G, A |
| 16879 | CKCS | EF | 77, 77 | 112, 112 | A, A |
| 16880 | CKCS | EF | 77, 77 | 112, 112 | G, G |
| 16888 | CKCS | CONTROL | 77, 77 | 106, 106 | G, G |
| 16891 | CKCS | EF | 77, 77 | 112, 112 | A, A |
| 16893 | CKCS | CONTROL | 77, 77 | 106, 112 | G, A |
| 16900 | CKCS | EF | 77, 77 | 106, 112 | G, A |
| 16901 | CKCS | EF | 77, 77 | 106, 106 | G, G |
| 16903 | CKCS | CONTROL | 77, 77 | 112, 112 | A, A |
| 16905 | CKCS | EF | 77, 77 | 112, 112 | A, A |
| 16908 | CKCS | EF | 77, 77 | 112, 112 | A, A |
| ***raw p values (31 EF cases, 38 controls)*** | | | **-** | **5.36 x 10^-13^** | **6.81 x 10^-11^** |
| ***raw p values (19 CKCSID cases, 38 controls)*** | | | **2.98 x 10^-22^** | **-** | **-** |
