# Supplementary material for: Parallel Mapping and Simultaneous Sequencing Reveals Deletions in BCAN and FAM83H Associated with Discrete Inherited Disorders in a Domestic Dog Breed
Source: PLoS Genet. 2012 Jan 12;8(1):e1002462. doi: 10.1371/journal.pgen.1002462 (PMC3257292; doi:10.1371/journal.pgen.1002462)
Supplement: Table S2 — Primers used for cDNA sequencing of the FAM83H and BCAN genes. (DOC) [file pgen.1002462.s005.doc]

**Table S2**

| **Forward primers** | | **Reverse primers** | | **Expected product** |
| --- | --- | --- | --- | --- |
| **Name** | **Sequence** | **Name** | **Sequence** | **(bp)** |
| BCAN_cDNA_1 | AGTGGAGAAAGGGGTTTTGTG | BCAN_cDNA_2 | CACTCAGCACCAGGGACAC | 582 |
| BCAN_cDNA_3 | GGTCAAGTGGACCTTCCTGTC | BCAN_cDNA_4 | CTGGGTCCACCACTCCATAGT | 500 |
| BCAN_cDNA_5 | TTCTCTACCGGGAAGGCTCT | BCAN_cDNA_6 | GGAAGCAGTACACGTTGAAGC | 585 |
| BCAN_cDNA_7 | AGTGTGCGCTATCCCATTGT | BCAN_cDNA_8 | GATGGCCTAGGCTGTAGGACT | 626 |
| BCAN_cDNA_9 | GTCCTCCGAAGAGGAAGACAA | BCAN_cDNA_10 | CTGAACCCGCACTGAGGT | 547 |
| BCAN_cDNA_11 | ACAGGGAGCTCTGAGGATAGC | BCAN_cDNA_12 | GCCATCTGACCACAAGAAGTC | 531 |
| BCAN_cDNA_13 | GGAGGAACAGGACTTCATCAAC | BCAN_cDNA_14 | CGTGGTCACTTCCTATGATGG | 628 |
| BCAN_cDNA_15 | GTAGACACGGTGCTTCGCTAC | BCAN_cDNA_16 | GGCTGGTTTTACTGGTTCTCC | 577 |
| FAM83H_F1 | CTCCTGCTGCCCCAGTCC | FAM83H_R1 | GCACTTCACCAAGCAGGTCCA | 588 |
| FAM83H_F3 | GGTACCTGCCACCTCACTACA | FAM83H_R3 | GTCAGCCATGTCCAGGAAGT | 530 |
| FAM83H_F4 | CTACTGGCCCATGAACTCAGA | FAM83H_R4 | CTCCTCATCGAAGCTGGAGAC | 538 |
| FAM83H_F5 | TCTGCCCAGCAGGTGGTG | FAM83H_R5 | GGTCGAGGAAAGAGGGGAATC | 586 |
| FAM83H_F6 | GTGGATTTCCTGCGCGTG | FAM83H_R6 | AAGGCGTCCATCTCCAGGT | 599 |
| FAM83H_F7 | GGAGCTACAGCTTCATGTGGT | FAM83H_R7 | TCTGAAAGCGCAGGTCGT | 594 |
| FAM83H_F8 | AAGCGGCACAGCTTCGCA | FAM83H_R8 | CATGGCAGCCGCTCAGGTAG | 556 |
| FAM83H_F9 | TGGACTACGTGCCGTCCAG | FAM83H_R9 | CGCTGAAGATCAGGGAGGAG | 543 |
| FAM83H_F10 | CGAGGCATACGAGGACGAC | FAM83H_R10 | AAAGGAGTCGCGCAGGTC | 571 |
| FAM83H_F11 | CCGAGCTCCTGGAGAAGTACA | FAM83H_R11 | GACTCCTCGGCGAAGGTAAG | 553 |
| FAM83H_F12 | AGGCACCTCACCCTGAGC | FAM83H_R12 | GATCTGCTCCAGAATGGCTTT | 500 |
| FAM83H_F13 | CAGCTGCTGAGCCCCAAG | FAM83H_R13 | ATGAACTTGCCCACCTTGCT | 599 |
| FAM83H_F13 | CAGCTGCTGAGCCCCAAG | FAM83H_R14 | GTTCTGCTGCCTGGTGTGAAG | 723 |
