# Supplementary material for: Parallel Mapping and Simultaneous Sequencing Reveals Deletions in BCAN and FAM83H Associated with Discrete Inherited Disorders in a Domestic Dog Breed
Source: PLoS Genet. 2012 Jan 12;8(1):e1002462. doi: 10.1371/journal.pgen.1002462 (PMC3257292; doi:10.1371/journal.pgen.1002462)
Supplement: Table S3 — Primers used for qPCR assays of the BCAN, FAM83H and ACTB (control) genes. All probes were 5′ 6-FAM and 3′ Iowa Black labelled, with internal ZEN labelling. (DOC) [file pgen.1002462.s006.doc]

**Table S3**

| **Assay name** | **Forward primer sequence** | **Probe sequence** | **Reverse primer sequence** | **size (bp)** |
| --- | --- | --- | --- | --- |
| BCAN | CACCGTGTCTACTTCATAGCG | CACAGGACACCAGCCCCATCTT | GAGTGATGTACCCTGCAACTAC | 133 |
| FAM83H | CCACGTGAAGGAGAAGTTTCTG | TGTAGCTCCCGCTCATCACCAC | TTCTCGAAGGACCACATGAAG | 81 |
| ACTB | CCAACCGTGAGAAGATGACC | CGAGACTTTCAACACCCCAGCCA | CGTACAGGGACAGCACAG | 90 |
