# Supplementary material for: Parallel Mapping and Simultaneous Sequencing Reveals Deletions in BCAN and FAM83H Associated with Discrete Inherited Disorders in a Domestic Dog Breed
Source: PLoS Genet. 2012 Jan 12;8(1):e1002462. doi: 10.1371/journal.pgen.1002462 (PMC3257292; doi:10.1371/journal.pgen.1002462)
Supplement: Table S4 — Primers used in the genotyping assay for the BCAN and FAM83H mutations. The expected mutant product size for the CKCSID primer pair refers to the 1 bp deletion in the FAM83H gene and for the EF primer pairs refers to the 15,724 bp deletion encompassing the first three exons of BCAN. (DOC) [file pgen.1002462.s007.doc]

Table S4

| **Forward primers** | | **Reverse primers** | | **Expected product**  **size (bp)** | |
| --- | --- | --- | --- | --- | --- |
| **Name** | **Sequence** | **Name** | **Sequence** | **Wild-type** | **Mutant** |
| CKCSID_F | 6Fam-CTTACACCCTGGCCCCGTA | CKCSID_R | GGTCGAGGAAAGAGGGGAAT | 135 | 134 |
| EF_F | 6Fam-TGTGCTCAGGAGTCTGTCCA | EF_bridge_R | GCAACCTACTCAGACCTGGAA | 15,831 | 115 |
| EF_F | 6Fam-TGTGCTCAGGAGTCTGTCCA | EF_normal_R | GGAGCAAATGCTCTGGAAGG | 105 | N/A |
